# Probe Capture Enrichment Methods for HIV and HCV Genome Sequencing and Drug Resistance Genotyping

**DOI:** 10.3390/pathogens11060693

**Published:** 2022-06-16

**Authors:** Chantal Munyuza, Hezhao Ji, Emma R. Lee

**Affiliations:** 1 National HIV and Retrovirology Laboratories, National Microbiology Laboratory at JC Wilt Infectious Diseases Research Centre, Public Health Agency of Canada, Winnipeg, MB R3E 3R2, Canada; chantal.munyuza@phac-aspc.gc.ca (C.M.); hezhao.ji@phac-aspc.gc.ca (H.J.); 2 Department of Medical Microbiology and Infectious Diseases, University of Manitoba, Winnipeg, MB R3E 0J9, Canada

**Keywords:** HIV, HCV, probe capture, enrichment, next-generation sequencing

## Abstract

Human immunodeficiency virus (HIV) infections remain a significant public health concern worldwide. Over the years, sophisticated sequencing technologies such as next-generation sequencing (NGS) have emerged and been utilized to monitor the spread of HIV drug resistance (HIVDR), identify HIV drug resistance mutations, and characterize transmission dynamics. Similar applications also apply to the Hepatitis C virus (HCV), another bloodborne viral pathogen with significant intra-host genetic diversity. Several advantages to using NGS over conventional Sanger sequencing include increased data throughput, scalability, cost-effectiveness when batched sample testing is performed, and sensitivity for quantitative detection of minority resistant variants. However, NGS alone may fail to detect genomes from pathogens present in low copy numbers. As with all sequencing platforms, the primary determinant in achieving quality sequencing data is the quality and quantity of the initial template input. Samples containing degraded RNA/DNA and/or low copy number have been a consistent sequencing challenge. To overcome this limitation probe capture enrichment is a method that has recently been employed to target, enrich, and sequence the genome of a pathogen present in low copies, and for compromised specimens that contain poor quality nucleic acids. It involves the hybridization of sequence-specific DNA or RNA probes to a target sequence, which is followed by an enrichment step via PCR to increase the number of copies of the targeted sequences after which the samples are subjected to NGS procedures. This method has been performed on pathogens such as bacteria, fungus, and viruses and allows for the sequencing of complete genomes, with high coverage. Post NGS, data analysis can be performed through various bioinformatics pipelines which can provide information on genetic diversity, genotype, virulence, and drug resistance. This article reviews how probe capture enrichment helps to increase the likelihood of sequencing HIV and HCV samples that contain low viral loads and/or are compromised.

## 1. Introduction

After four decades of intense efforts from all relevant fields across the world, HIV/AIDS remains a significant global public health concern. According to the Joint United Nations Programme on HIV/AIDS (UNAIDS), approximately 38 million people were living with HIV and an estimated 1.7 million people were newly infected with HIV in 2020 worldwide [1,2]. UNAIDS has set ambitious targets for the elimination of HIV/AIDS by 2030 [3]. The UNAIDS 95-95-95 targets stipulate that 95% of people living with HIV (PLWH) should be aware of their HIV status, 95% of people who are aware of their status should be receiving treatment, and 95% of people on treatment should be virally suppressed [3]. Likewise, hepatitis C virus (HCV) is another major bloodborne pathogen of significant public health concern. An estimated 58 million people currently live with chronic HCV infection, and approximately 1.5 million new HCV infections occur each year [4]. In 2016, the World Health Organization (WHO) developed the Global Health Sector Strategy on Viral Hepatitis. This strategy aims to treat 80% of HCV infections, reduce new viral hepatitis infections by 90%, and reduce deaths caused by viral hepatitis infection by 65% by 2030 [4]. HIV and HCV share commonalities in that both are enveloped viruses with a positive-sense, single-stranded RNA genome. In addition, both viruses are featured by their significant genetic diversity, resulting largely from their rapid replication rates and the error-prone reverse transcriptases they rely on [5,6,7]. Effective HIV and HCV strain and drug resistance monitoring facilitated by genome sequencing and drug resistance (DR) genotyping help monitor the progress towards these elimination targets. 

Conventional Sanger sequencing has been the primary technology applied in genome sequencing and genotypic DR testing for HIV and HCV [8,9]. Since 2005, next-generation sequencing (NGS) technologies have revolutionized the sequencing methodology, with significantly improved scalability, data throughput, sensitivity for minority resistant variants, and cost-effectiveness when batched sample testing is performed [10,11,12,13]. Nevertheless, the concentration and integrity of the input viral RNA or DNA templates determine the success of viral genotyping, regardless of the sequencing technology applied. Low viral load (VL) and low integrity often pose a significant challenge when sequencing samples collected from patients on antiviral therapy or those with severe RNA degradation [14,15]. 

Probe capture enrichment (also called target enrichment sequencing or hybridization capture) is a fairly recent methodology used to sequence samples containing low genomic copy numbers of a particular pathogen versus the host or from samples that have been compromised [16]. Hybridization capture involves the hybridization of sequence-specific DNA or RNA probes to a target fragment of DNA [17]. Probes are often custom-designed, targeting specific regions of interest within the template genome. For example, HIV probes can be designed to capture all major subtypes or to target particular subtypes such as subtype B of HIV-1 [18,19]. This method when performed prior to NGS would allow for complete genomes to be reconstructed directly from clinical samples. Whole genome sequencing data could then have various applications such as phylogenetics, epidemiology, and drug resistance testing [16]. Implementing this method in clinical diagnostic settings would have a direct effect on patient care as the information provided can guide patient treatment plans.

This promising method has been used successfully in a wide array of pathogens, including the parasite *Plasmodium falciparum* [20], fungi such as *Candida albicans* [21], bacteria such as *Mycobacterium tuberculosis* [22], and *Chlamydia trachomatis* [23] and viral pathogens such as HIV [18,19,24,25,26,27,28,29], HCV [24,28] and SARS-CoV-2 [30]; however, currently there is no consensus /standardized target enrichment protocol for HIV or HCV [16,31]. In this review, the various aspects of probe capture enrichment protocols used on HIV, and in some cases HCV, will be presented. 

## 2. Overview of Experimental Methods

**Figure 1:**
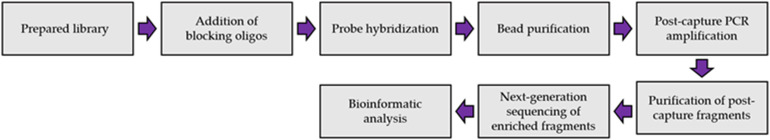
Overview of target-enrichment NGS procedure.

Hybridization capture protocols all include the same general steps [32]. The first step is nucleic acid extraction from a sample (DNA and/or RNA). This is followed by library preparation which will differ depending on the target organism, the quality and quantity of sample, and the library preparation kit being used. Target enrichment will occur after the library preparation. This process involves steps to hybridize the probes to the target sequence, enrich the probe-target complex, and elution to obtain the enriched fragment of interest. PCR-amplification will then be conducted to prepare the NGS library before sequencing on an NGS platform. The NGS data can then be processed using a professional bioinformatics platform for further analysis and alignment of the reads (Figure 1).

## 3. Extraction Method

Sequencing projects typically begin by extracting nucleic acid from a given sample. The steps involved in the extraction of DNA or RNA include cell lysis, removal of membrane lipids (or other nucleic acids), purification, and concentration of the nucleic acid [33]. The most common methodologies for nucleic acid extraction include full automation or manually conducted kits. Target enrichment protocols mainly use spin columns or an automated liquid-handling robot. These two nucleic acid extraction methods were evaluated for their advantages and disadvantages by N. Ali et al. [33]. They found that column-based nucleic acid extraction is one of the best techniques used as it is fast and its results are easily reproducible. The main drawback is that it requires a small centrifuge that can generate aerosols and lead to a slight chance of cross-contamination. Conversely, automated liquid handling robots offer precise handling of reagents and samples, reducing sample loss and artificial errors. However, the main drawback to this method would be the high cost of the equipment.

The nucleic acid extraction methods used in some target enrichment protocols are summarized in Table 1. Another consideration involved in nucleic acid extraction is the starting material. Nucleic acid extraction from whole blood, plasma, and serum is typically more successful than extraction from dried blood spots (DBS) [33,34,35]. With its easiness of sample collection and relieved requirements for transportation and storage, DBS is becoming a popular, cost-effective alternative to plasma, serum, or whole blood for HIV-1 genotyping and VL monitoring in resource-limited settings [15,36]. However, one primary limitation of DBS for such molecular assays is that the nucleic acid integrity can be significantly compromised, making downstream PCR amplification difficult [15,36]. Although further studies are warranted, the probe capture methodology could be a solution to salvage samples of poor viral RNA integrity for molecular assays. 

A successful library can be prepared from nucleic acid extracted from various sample types with either a manual or an automated protocol. Therefore, the primary considerations in choosing an extraction protocol for target enrichment will depend upon cost expectations and the availability of the required equipment.

## 4. Library Preparation Method 

**Figure 2:**
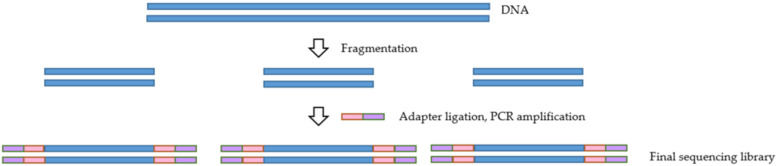
Overview of the library preparation process.

For any NGS-based project, the library preparation method is of utmost importance. The ability to generate a high-quality library is necessary for obtaining successful sequencing data. NGS library preparation is when the DNA fragments are prepared for sequencing via the addition of specific adapter sequences onto the ends of the DNA fragments (Figure 2) [37]. Several different library preparation kits and protocols can be used to produce a library, some of which are compiled in Table 2. While these kits may differ regarding their particular protocol and the amount of sample input required, most kits involve enzymatical or mechanical DNA fragmentation followed by tagmentation and incorporation of adapter sequences to the ends of the fragments. The derived libraries are then amplified and quantified prior to sequencing. 

The fragmentation step is vital to the target enrichment process as it influences its outcome. Shorter fragments are captured with higher specificity than longer pieces [38]. An additional consideration when selecting a library preparation kit for target enrichment is the number of PCR amplification steps. PCR amplification can introduce bias when DNA fragments are not all amplified with the same efficiency. A negative influence of PCR amplification on the uniformity of enrichment was noted in a study conducted by Mamanova et al. [38]. This negative influence was due to the bias introduced in PCRs before and after hybridization. 

Fragments that are either G-C rich or A-T rich are often underrepresented in the library preparations in comparison to G-C neutral fragments, which are amplified more efficiently [37]. One possible solution to this issue could be eliminating the PCR amplification step before hybridization, thus preventing the introduction of bias. However, while this may be possible when dealing with intact DNA available in large quantities, it lacks robustness in low-integrity samples [38]. As a result, this could be a concern when dealing with samples such as DBS, which may contain viral templates of low integrity, rendering the PCR amplification step inevitable. A mitigation solution in such cases could be to reduce the number of PCR cycles rather than remove the step entirely in order to reduce some of the bias while also generating a robust library from low integrity samples [38]. Additionally, Van Dijk et al. [37] have suggested several library preparation methods for reducing bias in NGS, including the use of Kapa HiFi polymerase instead of the standard Phusion polymerase used in Illumina library preparation.

## 5. Target Enrichment

**Figure 3:**
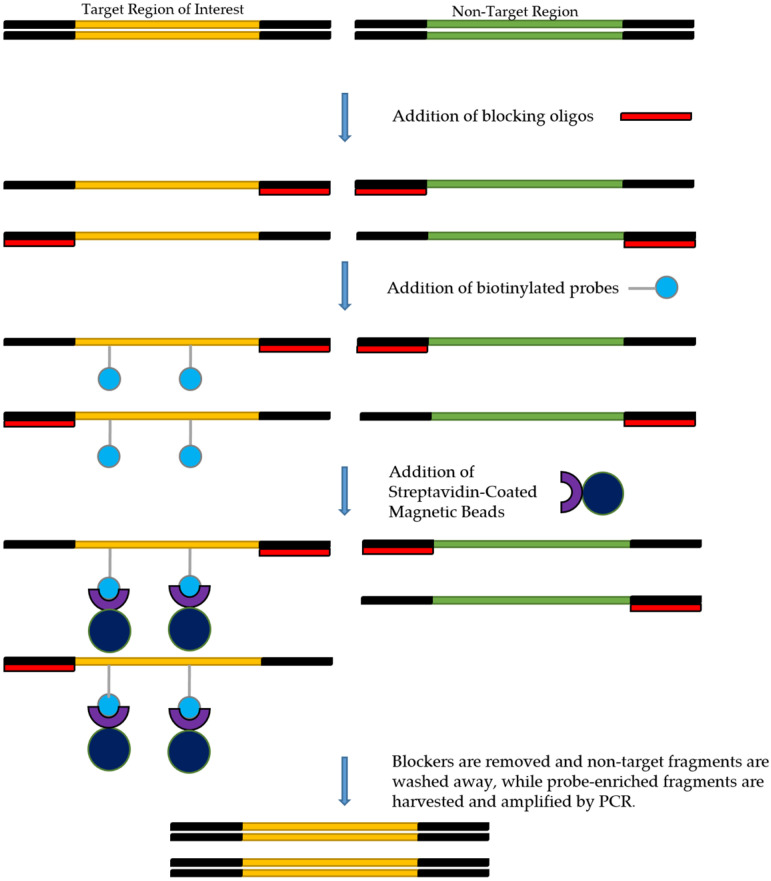
Overview of the target enrichment process.

Several target enrichment protocols, including xGen Lockdown probe protocol (IDT, Coralville, IA, USA), NimbleGen Seq Cap EZ system (Roche, Indianapolis, IN, USA), and the SureSelect Target Enrichment System (Agilent Technologies, Santa Clara, CA, USA), all operate using the same general procedure (Figure 3). However, the IDT xGen Lockdown probe protocol appears to be the most commonly used [39]. This protocol recommends using 500 ng of each prepared library as the input. Enrichment steps include combining DNA with the blocking oligos, after which the mixture is dried using a SpeedVac system. Blocking oligos are short oligonucleotide sequences that are added to decrease the possibility of hybridization between library adapters and capture probes during the target enrichment process [39]. The hybridization reaction can then be performed by combining the biotinylated probes with the dried DNA. Following hybridization, streptavidin-coated beads are added to pull down the probe-target complexes. Non-target fragments with no probe binding will then be washed off, and post-capture PCR amplification will follow to amplify the target fragment further. The final step involves purification of the post-capture PCR amplicons, after which the enriched library may be quantified and validated for sequencing on a NGS instrument. Some of the commercially available probe capture enrichment kits are listed in Table 3.

**Figure 4:**
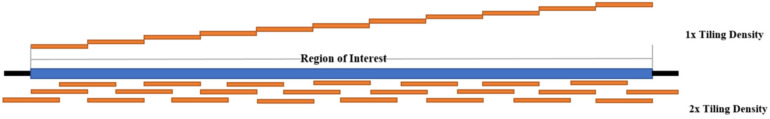
Comparison of 1× and 2× Tiling Density (Adapted from IDT).

The probes used in target enrichment are either DNA or RNA explicitly designed for the genomic region of interest. Probes are designed to the desired tiling density across the target region. The tiling density refers to the extent of the coverage of the target region by the probes. For example, 1× tiling density means that the probes cover the region of interest one time. In contrast, 2× tiling density means that the region of interest would be covered twice using a series of overlapping probes. Figure 4 depicts the differences between 1× and 2× tiling densities. The probes are often approximately 120 nt in length; however, this could differ, and are labeled by 5′ terminal biotinylation. Once the desired probes have been designed, they can then be synthesized by a biotechnology company for use in enrichment studies. Table 4 summarizes various probe design methods that have been used in reported target enrichment studies.

In studies focusing on a highly diversified virus such as HIV or HCV, probe design takes careful consideration if attempting to be inclusive of all subtypes and groups. In order to design probes to variable sequences such as those present in the different subtypes of HIV, one strategy is to first design the probes based on a consensus sequence and then subsequently design probes that will cover the variable regions for each subtype to be covered [19]. Alternatively, probes can be designed to be specific to one subtype rather than inclusive of all subtypes [26]. 

While the IDT xGen Lockdown probe protocol appears to be the most commonly used target enrichment protocol, an alternative protocol that has recently been gaining attention is the myBaits Hybridization Capture Kit by Arbour Biosciences (Ann Arbor, MI, USA). The myBaits protocol involves using pools of in-solution biotinylated RNA/DNA probes that are provided with reagents and allow for targeted sequencing on NGS platforms such as Illumina (San Diego, CA, USA), Ion Torrent, PacBio (Menlo Park, CA, USA), and Nanopore [42]. This kit also allows the user to use custom-designed probes with the kit.

The specific design of the probes will be influenced by the particular goal of the laboratory investigation. Additionally, the choice between RNA and DNA probes may depend on factors such as cost, storage requirements, and stability of the probes. A big advantage of using DNA probes is their stability as they can be safely stored at −20 °C, whereas RNA probes are sensitive to freeze-thaw cycles and need to be held at −80 °C for long-term storage [46]. RNA probes are often used due to the increased stability and hybridization efficiency of RNA-DNA duplexes compared to DNA-DNA duplexes [46].

## 6. Next-Generation Sequencing

After target enrichment, samples are sequenced on an NGS platform [47]. Although several NGS platforms are available, the MiSeq and NextSeq systems by Illumina have been most commonly used in target enrichment studies [19,25]. Both the MiSeq and the NextSeq operate using sequencing by synthesis technology in which the addition of fluorescently labeled nucleotides is tracked as the DNA chain is copied [47]. This process occurs in a massively parallel fashion, with the number of cycles determining the read length. The main difference between the two platforms is the read length and data output. MiSeq generates a maximum read length of 600 bp with a maximum output of 13.2–15 Gb compared to a maximum read length of 300 bp reads and output of 32.5–39 Gb with the NextSeq [48]. Both the MiSeq and NextSeq have been used successfully in target enrichment studies, so the choice of which sequencing platform to use will depend on the specifics of the research project itself and the availability of sequencing instruments.

## 7. Post-Sequencing Analysis (Bioinformatics)

After completion of sequencing on an NGS instrument, the data from the sequencing run should be analyzed using sophisticated bioinformatics tools. Both MiSeq and NextSeq systems provide read information in a fastq file, which can then be imported into bioanalytic software for analysis. Regardless of the platform, many researchers apply the same procedures to refine their sequencing data. This includes an initial data cleaning up by discarding reads of low quality scores. Adapter sequences are then removed from the reads. The remaining good quality reads are then mapped to a reference sequence available from GenBank or even a custom-defined reference. Once the reads have been aligned, a consensus sequence can be derived and the final alignment determined for further downstream applications [19,25,26]. A summary of the bioinformatics tools that have been used in target enrichment studies of HIV and HCV viruses can be found in Table 5.

## 8. Target Enrichment Performance

The success of target enrichment protocols has been demonstrated in studies comparing sequencing data from a run without enrichment and a run with enrichment prior to sequencing. In a study by P. Miyazato et al. [26], libraries prepared in the absence of enrichment resulted in 1.9% of the total reads mapping to the provirus. When the same libraries were enriched the total number of reads mapping to the provirus was increased from 1.9% to 99%. Similarly, in a study by S. Iwase et al. [25], DNA-capture sequencing was tested in HIV-1 infected latent cell lines. In the absence of target enrichment, from a total of 1.6 × 10^6^ reads, only three mapped to the provirus. This number increased in a subsequent experiment involving target enrichment prior to sequencing. In this case, out of 560,000 mapped reads, there were 28,000 reads aligning with the provirus [25]. This target enrichment protocol provided information that allowed researchers to characterize the provirus using a new method and authors indicated its applications to other experiments aiming to treat HIV-1 infection. 

In addition, target enrichment has also been shown in an HIV study by J. Yamaguchi et al. [19], to aid in the sequencing of low titer samples. They found that the genomes obtained from samples with VLs between log 4 and 5 copies/mL were still incomplete in the absence of the enrichment protocol procedure. In addition, when using samples at even a lower titer of log 3.5 copies/mL sequencing without the enrichment steps resulted in 20–50% coverage only. In comparison, sequencing the same low titer samples (log 3.5 copies/mL) using the enrichment protocol resulted in full genome sequences. This result is important as it indicates that low titer specimens, such as those present in patients undergoing antiretroviral therapy, may be characterized using the probe capture enrichment method.

## 9. Limitations

Despite the potential benefit target enrichment procedures could have in the study of highly diverse pathogens, like HIV and HCV, there are limitations to its implementation in a clinical diagnostic setting. A major drawback to using this method is the elevated cost of the target enrichment procedure which would make it difficult to implement in low-income settings. The estimated cost per sample from extraction to NGS is approximately $65 US although the cost may be lower if the probes are diluted, and a larger number of samples are pooled during hybridization [46]. In addition to the cost, target enrichment procedures often involve lengthy and complex protocols which would require skilled individuals who are knowledgeable about the various components of the protocols. These factors would make it difficult for a target enrichment procedure to be implemented in a clinical setting where results are required in a timely manner, especially in cases where novel pathogens are of interest [16]. 

## 10. Conclusions

Next-generation sequencing-based viral genome sequencing is crucial to understanding the ever-changing dynamics of HIV and HCV. The ability to generate quality sequencing data from samples with low viral titre or samples with poor nucleic acid integrity is important. Target enrichment has emerged as a potential solution to the problems of sequencing difficult samples and can potentially enable complete viral genome sequence even for low-quality clinical specimens. Increased adoption of such technology in research and development fields for HIV, HCV, and other pathogens is foreseeable.

## Figures and Tables

**Table 1:** Summary of nucleic acid extraction methods used in reported target enrichment protocols.

Virus	Samples Tested	Extraction Method	Ref
HIV	HIV-1 (plasma)	m2000sp RNA protocol (Abbott Laboratories)	[19]
HIV	HIV-1 (subtype B) from HIV-1-infected latent cell lines	DNeasy Blood and Tissue Kit (Qiagen)	[25]
HIV	HIV-1-infected cell line (ACH-2)	DNeasy Blood and Tissue Kit (Qiagen)	[26]
HCV	Clinical HCV samples (plasma)	NucliSENS Magnetic Extraction System (bioMérieux)	[24]
HIV	HIV-1 infected cell lines (ACH-2, J-Lat)	Gentra Puregene cell kit (Qiagen)	[27]
HCV	Clinical HCV samples (plasma), In vitro RNA transcipts, assay controls (plasma)	Agencourt RNAdvance blood kit (Beckman Coulter), QIAamp viral RNA minikit (Qiagen), NucliSENS magnetic extration system (bioMérieux)	[28]
HIV	Clinical HIV samples (plasma)	NucliSENS easyMAG system (bioMérieux)	[18]
HIV	Clinical HIV samples from peripheral blood mononuclear cells	EZ1 Virus Mini Kit v2.0 (Qiagen)	[29]

**Table 2:** Summary of library preparation methods used in target enrichment protocol.

Virus	Library Preparation Method	Ref
HIV	Nextera XT Kit (Illumina)	[19,29]
HIV	NEBNext UltraDNA II library preparation kit and NEBNExt multiplex oligos for Illumina (New England BioLabs)	[25,26]
HCV	NEBNext^®^ UltraTM Directional RNA Library Prep Kit for Illumina^®^ (New England Biolabs)	[24]
HIV	SPRI-TE nucleic acid extractor automated library preparation (Beckman Coulter) with NEXTflex adapters (Bioo Scientific)	[27]
HCV	KAPA Library Preparation Kit with index tagging using KAPA HiFi HotStart (KAPA Biosystems) and NEBNext multiplex oligos for Illumina Index Primer Sets 1 and 2 (New England BioLabs), SureSelect^XT^ Target Enrichment (Aligent), NEBNext Ultra Directional RNA Library Prep kit for Illumina (New England BioLabs)	[28]
HIV	SMARTer Stranded Total RNA-Seq Kit V2—Pico Input Mammalian (Clontech, Takara Bio)	[18]

**Table 3:** Commercially available probe capture enrichment kits.

Company	Kit	Compatible NGS Platforms	Type of Baits	Ref
Agilent Technologies	SureSelect^XT^ Target Enrichment System	HiSeq, MiSeq, NextSeq 500, NovaSeq 6000	Pre-designed or custom designed DNA probes	[40]
Agilent Technologies	SureSelect^XT^ RNA Target Enrichment System	HiSeq, MiSeq, NextSeq 500	Pre-designed or custom designed RNA probes	[41]
Arbor Biosciences	myBaits Hybridization Capture for Targeted NGS	Illumina platforms, Ion Torrent, PacBio, Oxford Nanopore Technologies	Pre-designed or custom designed RNA or DNA probes	[42]
Integrated DNA Technologies (IDT)	xGen™ NGS Hybridization Capture	Illumina platforms	Pre-designed or custom designed DNA probes	[43]
Lucigen	NxSeq HybCap Target Enrichment Kit	Illumina platforms, Ion Torrent	Custom designed RNA probes	[44]
Roche	NimbleGen Seq Cap EZ system	Illumina platforms	Pre-designed or custom designed DNA probes	[45]

**Table 4:** Summary of probe design methods used in target enrichment protocols.

Virus	Probe Design/Enrichment Method	Sequencing Platform	Ref
HIV	120 nt biotinylated DNA probes based on consensus sequences of HIV-1 and HIV-2 (xGen Lockdown probes and reagents, Integrated DNA Technologies)	MiSeq (Illumina)	[19]
HIV	120 nt biotinylated DNA probes based on HXB2 reference sequence (xGen Lockdown probes and reagents, Integrated DNA Technologies)	MiSeq or NextSeq (Illumina)	[25]
HIV	120 nt biotinylated DNA probes based on HXB2 reference sequence (xGen Lockdown probes, Integrated DNA Technologies) with SeqCap EZ Hybridization and Wash Kit (Roche NimbleGen)	MiSeq or NextSeq (Illumina)	[26]
HCV	120 nt DNA oligonucleotide probes (xGen Lockdown probes, Integrated DNA Technologies) and xGen^®^ Lockdown^®^ protocol (Integrated DNA Technologies)	MiSeq (Illumina)	[24]
HIV	120 nt DNA oligonucleotide probes (xGen Lockdown probes, Integrated DNA Technologies) with Dynabeads MyOne Streptavidin T1 (Life Technologies), PCR enrichment with Kapa HiFi DNA polymerase	MiSeq (Illumina)	[27]
HCV	120 nt RNA probes spanning 953 GenBank HCV reference genomes. Enrichment using xGen Lockdown protocol (Integrated DNA Technologies), NimbleGen Seq Cap EZ system (Roche), SureSelect Target Enrichment System (Agilent), or SureSelect^XT^ Target Enrichment (Agilent)	MiSeq (Illumina)	[28]
HIV	Custom HIV-specific biotinylated 120 nt probe set (XGen Lockdown Probes, Integrated DNA Technologies) with SeqCap EZ hybridization and wash kit (Roche)	MiSeq (Illumina)	[18]
HIV	Custom HIV-specific 120 nt probes (Arbor Biosciences) used with the myBaits target capture kit (Arbor Biosceinces)	MiSeq (Illumina)	[29]

**Table 5:** Summary of bioinformatics platforms used in target enrichment protocols.

**Virus**	Bioinformatic Platforms Used	Ref
HIV	CLC Genomics Workbench 9.0 (CLC Bio) for analysis of reads, phylogenetic analysis using SIMPLOT	[19]
HIV	In-house Pearl script for selection of paired-reads and cleaning of the reads, BWA-MEM algorithm for alignment to reference, Samtools program and Picard command line tools to remove multiply aligned reads and duplicates, final aligned files visualized with Integrative Genomics Viewer (IGV)	[25]
HIV	BWA-MEM algorithm for mapping, Picard tool for the removal of PCR duplicates, Strand NGS (Strand Life Science) for the visualization of mapped data, Low Frequency Variant Detection Tool (CLC Genomics Workbench 7.5 software, CLC Bio) for error correction	[26]
HCV	QUASR v7.01 & CutAdapt v1.7.1 for trimming sequences, Bowtie v2.2.4 for comparision to human reference, BLASTn database for screenning reads, Vicuna v1.3 & V-FAT v1.0 for de novo assembly, Mosaik v2.2.28 for mapping reads back to assembly, V-Phaser v2.0 for calling variants, V-Profiler v1.0 for examining intra-host diversity	[24]
HIV	BWA-MEM algorithm for alignment, sambamba for marking duplicate alignments, Gene SeT AnaLysis Toolkit for gene ontology analysis	[27]
HCV	FastQC, Tanoti, in-house resistance mutation tools, de novo assembly using MetAmos Genome mapping, assembly, and finishing using CLC Genomics Workbench, DAA analysis using in-house script QUASR v7.01 & CutAdapt v1.7.1 for trimming sequences, Bowtie v2.2.4 for comparision to human reference, BLASTn database for screenning reads, Vicuna v1.3 & V-FAT v1.0 for de novo assembly, Mosaik v2.2.28 for mapping reads back to assembly, V-Phaser v2.0 for calling variants, V-Profiler v1.0 for examining intra-host diversity	[28]
HIV	Kraken for processing raw sequences, Trimmomatic for trimming sequences, SPAdes, metaSPAdes for assembly into contigs, cd-hit-est for cluster generation, shiver for mapping reads, Kallisto for mapping reads with no contigs assembled, phyloscanner for identifying and removing contaminant reads, Stanford drug resistance tool for determining consensus and minority drug resisitance levels	[18]
HIV	CLC Genomics Workbench software (CLC Bio/Qiagen) for analysis of reads and assembly by mapping to the HIV genome (HIV-1 Strain HXB2) from GenBank	[29]

## Data Availability

Not applicable.
